# Professional Prospects and Anticipations

**Published:** 1917-01-06

**Authors:** 


The Workers' Newspaper of Administrative Medicine and Institutional Life, Administration, National Insurance and Health.

No. 1596, Vol. LXI. SATURDAY, JANUARY 6, 1917. PRICE ONE PENNY

### PROFESSIONAL PROSPECTS AND ANTICIPATIONS.

The New Year is born in circumstances which in a degree much higher than is usual invite an endeavour to anticipate the future and to arrange plans for its due ordering and reception. The note of change and development is in the air and there is an acute consciousness that it will touch many things. Indeed, the feeling is that few things will escape it, and that institutions and organisations of all sorts and kinds will be called upon to answer for their qualities and to justify, if this may be, their existence and arrangements. The profession of medicine, like its fellows, is subject to the summons, and there are many happenings which show that public opinion is organising a judgment in this direction. Events which engage universal attention have set the profession in a position of such prominence relative to the national welfare that a verdict of some kind is inevitable, and the materials necessary for this verdict are undoubtedly being collected and scrutinised. What has been done for our Armies in the field, and what is still being done, both at home and abroad, for sick and wounded soldiers, are records which need fear no light. By universal admission the Medical Services of the Navy and Army have deserved well of the country, and the recognition of this truth can boast both an official and a popular endorsement. The haste of events, however, is one of the prominent features of the day and hour. No man and no organisation can find sufficient justification in a task which has been successfully accomplished so long as further duties await achievement. The claim is to continue in well-doing, and, even more than this, to rise to the new events which a new day announces. There is yet a further demand, and it is that, so far as is possible, these new events shall be foreseen and due provision made to meet them. Such are the standards which the present time imposes, and in a direct or an indirect fashion work will be judged by them. The medical profession, as others, must prove itself ready for the test.

What is most urgent at the moment seems to be an increase in the number of medical officers for the Army, and the difficulty is to provide these without prejudice to the necessary and legitimate wants of the civil population. It has been said that the Army authorities do not distribute effectively the men they have, and, judged by civilian standards, the criticism does not lack support. But in the present stress of affairs the official judgment must be largely trusted, and anything like obstruction or opposition will be hard to justify. Certainly one of the difficulties is the unequal distribution of the doctors in civil practice. It is beyond question that while in many industrial districts there is but a scanty supply, there are residential areas where medical practitioners abound. And, speaking generally, it is in the former that are found the younger men—that is, the men who are specially qualified for active military service. Yet for this service numbers of them cannot be taken for the simple but sufficient reason that their enlistment would mean the absence of a due Medical Service for the industrial classes, including the workers in munitions. These are the circumstances which have prompted the suggestion that the whole profession should be mobilised, that each man should hold himself ready to be sent where his work would be of most service to the nation. The proposal is in all conscience a serious one from the point of view of individual liberty, but in principle, at all events, it has been received with a minimum of protest, and in a number of official quarters it is receiving energetic support. The sole objection advanced has been less against the principle than against the application of it to the medical profession apart from other citizens. Whether mobilisation should or should not be applied to all citizens is hardly for discussion here, and we doubt whether, in existing circumstances, any high-and-dry constitutional argument is likely to be listened to with patience. If the national cause needs some special discipline of the medical profession we are sure that the profession will not stand aside merely because other classes do not come under the same pressure. Whatever may be the inconvenience to individuals the national purpose has to succeed, and those who are specially capable of serving it are happy in their opportunity. The profession is certainly not wanting in patriotism, and when the way is shown volunteers will hardly be wanting. The immediate task seems to be a scheme of organisation developed and applied by the profession itself, and signs are not wanting that this is fully realised. (*See also* "At the Close of the War," p. 277.)

